# Review of Hyperuricemia as New Marker for Metabolic Syndrome

**DOI:** 10.1155/2014/852954

**Published:** 2014-02-16

**Authors:** Laura Billiet, Sarah Doaty, James D. Katz, Manuel T. Velasquez

**Affiliations:** Department of Medicine, The George Washington University, 2150 Pennsylvania Avenue, NW, Washington, DC 20037, USA

## Abstract

Hyperuricemia has long been established as the major etiologic factor in gout. In recent years, a large body of evidence has accumulated that suggests that hyperuricemia may play a role in the development and pathogenesis of a number of metabolic, hemodynamic, and systemic pathologic diseases, including metabolic syndrome, hypertension, stroke, and atherosclerosis. A number of epidemiologic studies have linked hyperuricemia with each of these disorders. In some studies, therapies that lower uric acid may prevent or improve certain components of the metabolic syndrome. There is an association between uric acid and the development of systemic lupus erythematosus; the connection between other rheumatic diseases such as rheumatoid arthritis and osteoarthritis is less clear. The mechanism for the role of uric acid in disorders other than gout is not well established but recent investigations point towards systemic inflammation induced by urate, as the major pathophysiological event common to systemic diseases, including atherosclerosis.

## 1. Introduction

More than 150 years ago, Garrod observed that uric acid is elevated in the blood of subjects with gout [1]. However, ultimate proof that uric acid is a causative factor in gout was not forthcoming for another 100 years when it was demonstrated that intra-articular injection of sodium urate produces acute arthritis [2]. Currently, it is well appreciated that chronic hyperuricemia enhances deposition of uric acid in tissues other than the joints, leading ultimately to tophi, nephropathy, and kidney stones.

## 2. Hyperuricemia Is Not Limited to Gout

In recent years there has been a renewed interest in hyperuricemia and its association with a number of clinical disorders other than gout, including hypertension, atherosclerosis, cardiovascular disease, and chronic kidney disease. Indeed, hyperuricemia is commonly part of the cluster of metabolic and hemodynamic abnormalities including abdominal obesity, glucose intolerance, insulin resistance, dyslipidemia, and hypertension all often subsumed under the term “metabolic syndrome” [3, 4]. Not only collectively, but also individually, hypertension, obesity, dyslipidemia, hyperglycemia, and insulin resistance are positively correlated with serum levels of uric acid [5–9].

## 3. Hyperuricemia and Metabolic Syndrome and Its Components

The association between uric acid and metabolic syndrome is robust throughout human development. Epidemiological studies have demonstrated a close relationship between serum uric acid (SUA) levels and the presence of metabolic syndrome (and several of its components) among children and adolescents as well as adults [10]. Some studies have even noted the strong association between SUA and carotid atherosclerosis among obese children [10, 11]. One study analyzed the cross-sectional data of 1,370 US children and adolescents aged 12–17 years from the National Health and Nutrition Examination Survey (NHANES) 1999–2002 and found a graded positive association between SUA and the prevalence of metabolic syndrome or its components, independent of classical risk factors. They found that of the five components of metabolic syndrome, SUA was significantly associated with abdominal obesity, hypertriglyceridemia, and hyperglycemia; there was only a borderline association observed between SUA and high blood pressure.

Another compelling evidence suggests that hyperuricemia predicts the development of hypertension, obesity, and type II diabetes mellitus [12, 13]. A prospective study from the Framingham Heart Study original (*n* = 4883) and offspring (*n* = 4292) cohorts showed that individuals with higher serum uric acid, including younger adults, had higher future risk of type 2 diabetes.

Several studies conducted since the early 1990s have shown that increased serum uric acid precedes the development of hypertension. Feig, in studies of hypertensive adolescents, reported that an elevated uric acid over 5.5 mg/dL was observed in 90% of adolescents with newly diagnosed primary disease [14]. Specifically, there exists a strong linear correlation between serum uric acid and systolic blood pressure. The same group conducted a double-blind, placebo-controlled crossover study including 30 hypertensive adolescents who were randomized to allopurinol or placebo for 4 weeks. While 86% of the intervention group became normotensive following a decrease of uric acid below 5 mg/dL, this rate was only 3% in the control arm [15]. Another intervention study conducted in asymptomatic hyperuricemic patients documented benefits with allopurinol treatment in terms of blood pressure and C-reactive protein (CRP). In a landmark animal study by Mazzali et al. pharmacologically induced mild-to-moderate hyperuricemia via oxonic acid administration in rats resulted in the development of hypertension. Remarkably when uric acid elevation was prevented by treating with allopurinol or with a uricosuric agent, the development of hypertension could be abrogated [16]. This study supports the hypothesis that there exists a direct causative role for uric acid in the development of hypertension. Collectively, these data strongly suggest that uric acid may have a pathogenic role in the development of metabolic syndrome and associated cardiovascular disease, rather than simply functioning as a surrogate marker for such disorders.

## 4. Hyperuricemia in Rheumatic Diseases Other Than Gout

An association between hyperuricemia and rheumatic diseases, besides gouty arthritis, has been observed in epidemiologic studies. For example, in a study of 7374 adult women (age >20 yrs) in the Third National Health and Nutrition Examination Survey (NHANES), it was shown that women self-reporting RA had significantly higher SUA levels compared to women not self-reporting RA. In this study, predictors of SUA levels, besides self-reporting RA, were race/ethnicity, being married, smoking, use of alcohol, high body mass index, high C-reactive protein, elevated diastolic or systolic blood pressure, and increased glomerular filtration rate [17]. Furthermore, among patients with RA, one study has shown that SUA levels are correlated with the risk of renal dysfunction. SUA, independent of GFR, age, systolic blood pressure, total cholesterol, triglycerides, and RA duration, was determined to be a risk factor for kidney disease [18].

Similarly, Yang et al. demonstrated that SUA levels can be used to predict the development of renal disease in patients with SLE. The authors found that SUA is independently associated with the development of lupus nephritis in SLE patients [19].

There are also reports showing an association between uric acid and osteoarthritis (OA). One study in OA patients with hip replacement found that elevated SUA concentrations were associated with the presence of multijoint hand OA [20]. Another study has shown the occurrence of gout attacks in joint sites that demonstrated radiographical OA such as big toe, midfoot, knee, and distal finger joints. It is possible that OA may facilitate the localized deposition of urate crystals [21].

More recently, Denoble et al. assessed synovial fluid UA concentration in patients with knee OA but with no clinical signs for, or self-report of, gout and found that synovial fluid UA concentrations correlated positively with radiographic and scintigraphic measures of OA severity and synovial fluid interleukins (IL-18 and IL-1*β*) [22].

These findings suggest that synovial fluid UA is a risk marker for OA severity. Furthermore, they show a correlation between synovial fluid UA and IL-18 and IL-1*β*, two cytokines known to be produced by uric acid-activated inflammasomes. The association of synovial fluid IL-18 with OA progression also suggests the possibility that UA is a risk factor promoting the pathological process of OA (possibly through activation of the inflammasome).

## 5. Biochemistry and Biological Actions of Uric Acid

Uric acid is the end product of purine metabolism in humans and is generated by the action of the enzyme, xanthine oxidase (XO), which catalyzes the last two steps of uric acid conversion: hypoxanthine to xanthine and from xanthine to uric acid.

For many years, uric acid was regarded as a metabolically inert substance. However, there is ample evidence that uric acid has multiple actions impacting cellular metabolism. For example, uric acid can act as an endogenous antioxidant and a powerful scavenger of single oxygen peroxyl (ROS) and hydroxyl radicals (OH) [23]. Uric acid reacts with peroxynitrite and stabilizes endothelial nitric oxide synthase (eNOS) activity [24]. Its chemical limitations include the fact that uric acid cannot scavenge superoxide and that the presence of ascorbic acid in the plasma is required for its antioxidant effect. Finally, uric acid paradoxically behaves as a prooxidant and proinflammatory factor. A few points should be emphasized to better understand this apparent contradiction. First, uric acid functions differently inside cells versus the extracellular milieu, where it is present in soluble form [25]. Although it is a potent antioxidant in extracellular fluid, uric acid exerts prooxidative effects once inside the cell [26]. This effect is mediated by a NADPH oxidase-dependent pathway. It has been demonstrated that plasma uric acid is a circulating marker of oxidant damage in a variety of pathological conditions, including, among others, ischemic liver injury, ischemic-reperfusion injury, hyperlipidemia, chronic heart failure, atherosclerosis, and diabetes [27–31]. Thus, inhibition of XOR by allopurinol or other XOR inhibitors would be expected to reduce not only uric acid production but also NADH, which results in a favorable shift of NAD/NADH ratio.

## 6. Hyperuricemia and Atherosclerosis

Serum uric acid levels have an association with surrogate markers of atherosclerosis in a number of studies. Surrogate markers of atherosclerosis shown to have an association with hyperuricemia include carotid intima-media thickness (C-IMT) [11, 32–36], ankle brachial index [34], coronary artery calcification [37], and brachial-ankle pulse wave velocity (baPWV) [38]. In addition, many studies suggest an independent effect of elevated serum uric acid on atherosclerosis as measured by these surrogate markers after adjusting for the influence of metabolic syndrome and other factors [11, 33, 35, 39]. In particular, there is evidence that uric acid has direct effects on key processes involved in endothelial function and vascular remodeling [40].

Xanthine oxidoreductase has two forms. In physiologic conditions, it exists primarily as xanthine dehydrogenase, which has greater affinity for oxidized nicotinamide adenine reductase (NAD+) compared to oxygen. Under ischemic conditions, xanthine dehydrogenase is converted to xanthine oxidase. Xanthine oxidase uses oxygen as an electron acceptor instead of NAD+ resulting in the formation of superoxide anion and hydrogen peroxide alongside uric acid. It is unclear whether the resulting inflammation and arterial wall damage associated with hyperuricemia are related to the free radicals formed or to the uric acid itself [41].

As noted above, uric acid has both prooxidant and antioxidant activity. When acting an antioxidant, it chelates metals and scavenges oxygen radicals [42]. As a prooxidant, uric acid oxidizes lipids [43], reduces nitric oxide availability in endothelial cells [44], and increases reactive oxygen species [45]. Furthermore, as a prooxidant, high levels of serum uric acid cause increased lipid oxidation [43]. The resultant inflammation would be expected to disrupt reverse cholesterol transport, a function that is important to reduce cardiovascular risk [46].

Oxidants also cause endothelial dysfunction by reacting with and removing NO, thereby preventing vasodilation of the endothelium. Decreased NO and increased reactive oxygen species may promote a proinflammatory state that causes endothelial dysfunction and contributes to atherosclerosis and cardiovascular disease [47]. Finally, uric acid inhibits endothelial cell proliferation and stimulates C-reactive protein production in endothelial cells [48].

At the level of vascular smooth muscle cells, uric acid stimulates the production of monocyte chemoattractant protein-1 (MCP-1), a key chemokine implicated in atherosclerosis and chronic kidney disease. Increased production of MCP-1 by uric acid appears to be mediated via activation of mitogen-activated protein (MAP) kinase and cyclooxygenase-2, leading to increased cell proliferation and production of CRP and other inflammatory mediators [48]. Taken together, it is not surprising that several studies show that elevated serum uric acid levels are predictive of atherosclerosis or cardiovascular disease. For example, hypertensive hyperuricemic patients have higher levels of BMI, C-IMT, fasting plasma glucose, UA, hs-CRP, and proteinuria as compared with hypertensive normouricemic subjects. Hyperuricemia, *per se*, was found to be an independent predictor for atherosclerosis in patients with hypertension [33]. In another clinical study, serum uric acid was found to be associated with subclinical atherosclerosis in men with type II diabetes mellitus [34, 49]. Serum uric acid levels were also higher in patients with rheumatoid arthritis (RA) and cardiovascular disease than in those without cardiovascular disease despite the fact that RA is not usually associated with high serum uric acid levels [50]. Such findings do not appear to be limited by gender. In women with systemic lupus erythematosus, serum uric acid was found to be associated with arterial stiffness and of potential use as an indicator of subclinical atherosclerosis, although it was not independent of age and homocysteine levels [51], nor is it likely the situation that an absolute level of uric acid elevation is necessary for injurious outcome. This was seen in a population of psoriatic arthritis patients with subclinical atherosclerosis [52]. Of note, most of the patients in this study had serum uric acid levels that were in the normal reference range even though there remained a link between subclinical atherosclerosis and serum uric acid concentration [52]. This may mean that the reference ranges for patients with chronic inflammatory diseases may need to be adjusted. At the very least, elevated levels of serum uric acid, even if within the reference range, may need to be considered more carefully in populations at higher risk for cardiovascular disease.

In summary, while it may be true that, under normal physiologic conditions, uric acid functions as an antioxidant and protects against atherosclerosis, it appears that, under ischemic conditions, it becomes a prooxidant and increases the risk of atherosclerosis [41].

Hence, it is as yet unclear what the role of lowering uric acid may be in reducing cardiovascular disease risk. Further study is needed to show if lowering serum uric acid will also lower the incidence of atherosclerosis and cardiovascular disease.

## 7. Urate as a Mediator of Inflammation 

We now turn our attention to the involvement of uric acid in systemic inflammation: a central pathophysiological feature common to many forms of chronic noninfectious disorders such as obesity, hypertension, chronic heart failure, and cardiovascular disease, as well as generalized atherosclerosis. Indeed, there are epidemiological studies showing a relationship between serum uric acid and markers of systemic inflammation. In several population-based studies [53] in healthy men and women, serum uric acid is associated positively with CRP [54]. Similarly, serum uric acid is positively associated not only with CRP, but also with IL-6 and TNF-*α*, as reported in a study of 957 elderly individuals in Italy [55]. Moreover, serum uric acid predicted CRP increase during follow-up assessment [30]. In another population-based study conducted in Switzerland that included 6085 Caucasians aged 35 to 75 years, serum uric acid was found to be positively associated with CRP, TNF-*α*, and IL-6 (in both men and women) [56]. In patients with chronic heart failure, SUA is associated positively with TNF-*α* and IL-6. And in patients with metabolic syndrome, several components of the syndrome, including serum uric acid levels, are strongly correlated with serum levels of CRP, IL-6, and fibrinogen, again, consistent with high inflammatory activity [57]. Finally, there is experimental evidence that suggests that uric acid can directly stimulate the release of TNF-*α*. Specifically, infusion of soluble uric acid into mice produces a marked increase in circulating TNF-*α* levels [58]. Studies by di Giovine et al. have demonstrated that urate crystals stimulate production of TNF-*α* from human blood monocytes and synovial cells [59]. Along similar lines, cell culture experiments by Bordoni et al. have highlighted the role of uric acid in inflammation by demonstrating its role in signal transduction within the apoptotic pathway and also confirming that uric acid stimulates mononuclear cells to produce TNF-*α* [60].

Recently, Kono and coworkers, in a series of *in vivo* studies, demonstrated that uric acid plays a key role in the inflammatory response to necrotic cells in mice. These investigators found that dead cells not only release intracellular stores of uric acid but also produce it in large amounts postmortem (as nucleic acids were degraded) [61]. Using a newly developed transgenic (Tg) mouse that has reduced levels of uric acid, they further showed that uric acid depletion substantially reduces the cell death-induced inflammatory response. Similar results have been obtained with pharmacological treatments that reduced uric acid levels by either blocking its synthesis or hydrolyzing it in the extracellular fluids. Additionally, uric acid depletion selectively inhibits the inflammatory response to dying cells but not to microbial molecules or sterile irritant particles. Taken together, these data indicate that uric acid acts as an endogenous proinflammatory molecule released from dying cells as a result of tissue damage.

Recently, a critical role of intracellular inflammasomes (such as NALP3) has been identified. Innate immune complexes known to be responsible for triggering inflammation in response to danger-associated molecular patterns (DAMPs) have been implicated in MSU crystal associated inflammation [62, 63]. The inflammatory effect of MSU crystals appears to be mediated to a large extent by the NLRP3 inflammasome that drives IL-1*β* and IL-18 production. IL-1*β* is likely the main agent to trigger systemic inflammation, promoting extensive neutrophil infiltration and tissue restructuring [64]. Further evidence for a role of UA in inflammasome activation is the finding that inflammasome activation by pure synthetic hemozoin is reduced by allopurinol [65].

## 8. Urate and Innate and Adaptive Immunity

Uric acid is a danger signal that alerts the immune system to cell injury and triggers both innate and adaptive immune responses. It has been shown that dying cells injected into animals along with an antigen create a strong adjuvant effect and significantly increase the immune response to the initial antigen. Microcrystalline UA released from injured cells may be the danger signal triggering this adjuvant effect through the stimulation of CD8+ T cells [66].

Studies by Liu-Bryan et al. also implicate innate immune cellular recognition of naked MSU crystals by specific Toll-like receptors (TLRs) as a major factor in determining the inflammatory potential of MSU crystal deposits [67]. Recently it was shown that UA crystals in the presence of NF-*κ*B signaling were capable of stimulating dendritic cells to promote the release of cytokines associated with Th17 polarization indicating, a novel role for UA in driving proinflammatory Th17, suggesting that sterile inflammation modulates adaptive immunity, in addition to influencing early innate responses [68].

In summary, evidence has emerged that points towards a new interpretation of the pathophysiological role of uric acid. No longer can we view it simply as a phenomenon restricted to gout, but rather as an active agent involved in systemic inflammatory and innate immune responses. It is becoming clear that uric acid is not only a marker of the metabolic syndrome and associated cardiovascular risk factors, but also an agent provocateur of systemic inflammation and the innate immune response that contributes to the development and pathogenesis of cardiovascular disease. Indeed, it may be a key measure of blood vessel inflammation. This has implications not only for the metabolic syndrome (and accelerated atherosclerosis) but likely also for autoimmune vascular diseases such as vasculitis. Therefore, it is not surprising that patients with SLE and metabolic syndrome have significantly raised serum uric acid [69].

In short, an inflammatory microangiopathy may be seen in patients with connective tissue disease and uric acid may be an active participant in the process. It may be that physiologic signal transduction mediated by reactive oxygen species (ROS) along with the possibility of rapid XD to XO conversion plays a role as a trigger for the microvascular inflammatory response to clinical states of vascular injury [70].
